# Implementation of a Pilot-Scale Biotrickling Filtration Process for Biogas Desulfurization under Anoxic Conditions Using Agricultural Digestate as Trickling Liquid

**DOI:** 10.3390/bioengineering10020160

**Published:** 2023-01-25

**Authors:** Alejandra Lenis, Martín Ramírez, José Joaquín González-Cortés, Kristoffer Ooms, Johannes Pinnekamp

**Affiliations:** 1 Research Institute for Water Management and Climate Future, RWTH Aachen University, (FiW) e. V., Kackertstraße 15-17, D-52056 Aachen, Germany; 2 Department of Chemical Engineering and Food Technology, Cadiz University, 11510 Puerto Real, Spain

**Keywords:** hydrogen sulfide, anoxic desulfurization, biotrickling filter, denitrification

## Abstract

A pilot-scale biotrickling filter (BTF) was operated in counter-current flow mode under anoxic conditions, using diluted agricultural digestate as inoculum and as the recirculation medium for the nutrient source. The process was tested on-site at an agricultural fermentation plant, where real biogas was used. The pilot plant was therefore exposed to real process-related fluctuations. The purpose of this research was to attest the validity of the filtration process for use at an industrial-scale by operating the pilot plant under realistic conditions. Neither the use of agricultural digestate as trickling liquid and nor a BTF of this scale have previously been reported in the literature. The pilot plant was operated for 149 days. The highest inlet load was 8.5 gS-H_2_Sm^−3^h^−1^ with a corresponding removal efficiency of 99.2%. The pH remained between 7.5 and 4.6 without any regulation throughout the complete experimental phase. The analysis of the microbial community showed that both anaerobic and anoxic bacteria can adapt to the fluctuating operating conditions and coexist simultaneously, thus contributing to the robustness of the process. The operation of an anoxic BTF with agricultural digestate as the trickling liquid proved to be viable for industrial-scale use.

## 1. Introduction

Biogas is the most relevant product resulting from the anaerobic digestion of organic matter [1]. During the anaerobic digestion of agricultural waste, a composition of approx. 60 Vol.% methane (CH_4_) and 40 Vol.% carbon dioxide (CO_2_), with trace amounts of the highly toxic and corrosive hydrogen sulfide (H_2_S) gas, can be found [2]. Biogas is mainly used for electricity and heat generation, which can demand a high purity level, depending on the application. For gas grid injection in Germany, for example, H_2_S values smaller than 5 ppm_V_ are necessary [3]. The single most important biogas purification step prior to biogas utilization is the elimination of hydrogen sulfide (H_2_S), also known as desulfurization [4]. Desulfurization is usually carried out through chemical, physical or biological separation processes. Due to the consumption of precipitants and adsorbents, the physical and chemical desulfurization processes are usually associated with high operating costs. Chemical desulfurization processes include amine or caustic scrubbers, while physical desulfurization processes include water scrubbing or adsorption on activated carbon [5,6,7]. Commonly used biological desulfurization methods are based on the oxidation of H_2_S with an electron acceptor, which is usually oxygen (O_2_). The biological reaction of H_2_S with O_2_ enables almost a complete removal of H_2_S while allowing the formation of elemental sulfur and sulfate ions [8]. One of the most widespread biological desulfurization methods is direct aeration into the digester, which involves the feeding of air into the gas space of the anaerobic digester. This practice, though cost efficient and easy to maintain, carries some disadvantages. It poses a danger, since an explosive atmosphere can be built by mixing biogas with air. The specific placement of the air inlet in the digester is also not easily identifiable, resulting in an almost uncontrollable air feed [3].

Other commonly used processes are biotrickling filters (BTFs) and bioscrubbers (BS) [9]. Both BTFs and BS are implemented as a separate process unit, into which air is also fed. BS are characterized by two process units: an absorption column, where H_2_S is absorbed into the trickling liquid, and a separate tank bioreactor, where H_2_S is degraded and the trickling liquid regenerated [10]. While the aerobic operation of BS is the most common and is also commercially available, the desulfurization of biogas in BS under anoxic conditions has also been reported in the literature [11]. In BTF, the biogas passes through a column filled with carrier material colonized by microorganisms that degrade the H_2_S [12]. Although BTFs and BS are designed for direct air entry, which allows for better control of the air flow, the danger of an explosive atmosphere remains. Biological desulfurization by means of biofiltration using oxygen as an electron acceptor agent has been extensively researched and industrially implemented [13,14,15]. The biggest disadvantage of biological oxidative processes that use oxygen as the oxidizing agent is that the addition of air into the biogas stream aggravates the upgrading process of biogas to biomethane, due to the complicated removal of nitrogen [3]. Therefore, such purification processes are not utilized when upgrading biogas to biomethane.

Since the anoxic desulfurization of biogas does not require oxygen, no explosive atmosphere or aggravation of biogas upgrading occurs [16]. For these reasons, the research and implementation of the anoxic desulfurization in BTFs using nitrate (NO_3_^−^) as the electron acceptor instead of oxygen has been receiving increasing attention in recent years [13,14].

Most anoxic desulfurization studies were carried out on laboratory scale BTFs, with synthetic nutrient solutions and biogas mimic mixtures (N_2_ + H_2_S), as well as previously cultivated bacterial cultures. Although some studies were carried out with activated sludge from wastewater treatment plants or landfill leachate at pilot-scales [16,17,18,19] and have demonstrated a high removal efficiency, none have been carried out with agricultural digestate as trickling liquid. In this study, no inoculation of lab-cultivated bacteria was carried out. Instead, real agricultural digestate as both inoculum and nutrient medium, as well as trickling liquid in the absorption column, was used. This study analyzes the performance of a pilot-scale BTF for the desulfurization of biogas under anoxic conditions. The scope of this study was to investigate the role of nitrate levels in determining the capacity and efficiency of H_2_S elimination from biogas. Furthermore, no temperature or pH regulation was undertaken, in order to test the robustness of the complete process and propose a simplified approach to biogas desulfurization. The biogas was obtained directly from the air space of an agricultural fermentation plant, and its digestate was used as trickling liquid. This approach has several advantages compared to the previously mentioned chemical or physical methods. Using the digestate produced in a biogas plant as a trickling liquid can represent an economical benefit by reducing operational costs, especially compared to the use of amines or activated carbon, which have to be constantly replaced. Another advantage is that the digestate, which is generally used as a fertilizer for crops, will contain a higher level of sulfur, sulfate and dissolved H_2_S after biogas desulfurization in the BTF. H_2_S has been shown in the literature to have a positive impact on seed germination [20], thus simultaneously contributing to the digestate´s fertilizing effect and to a circular sulfur use.

The goal of studying the process under real conditions is to generate a solid foundation for its implementation on an industrial full-scale level. The operation of the pilot plant was therefore exposed to abrupt and unexpected changes in conditions, showcasing realistic industrial operation circumstances. To the author´s knowledge, this approach has not been reported in the literature until now.

## 2. Materials and Methods

**Figure 1:**
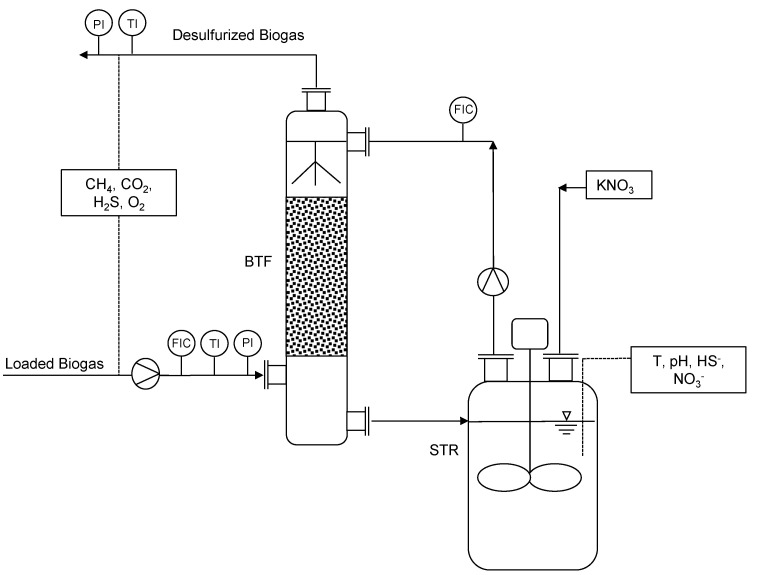
Pilot plant design with specification of flow indicator control (FIC), pressure and temperature indicator (PI, TI).

Two major process units made up the pilot plant; a BTF and a separate stirred tank reactor. The experimental set-up is shown in Figure 1. The BTF (163 L_total_, 88 L_packing bed_) filter is made of transparent PVC and was filled with cylindrical plastic beads also made out of PVC (755 m²m^−^³, 9.5 mm length, 3.5 mm radius, EvU Innovative Umwelttechnik GmbH, Gröditz, Germany) as packing material. The stirred tank reactor (1 m³) made of stainless steel was hydraulically coupled with the bottom part of the BTF. This serves as a regeneration step for the digestate, where samples of the digestate are taken in order to monitor pH, temperature, SO_4_^2−^ and N-NO_3_^−^. The stirred tank reactor and the BTF were designed by the authors and manually built by EvU Innovative Umwelttechnik GmbH in Gröditz, Germany. Temperature and pH are measured by means of a potentiometric sensor (eCHEM TpH-D, TriOS GmbH, Rastede, Germany). Sulfate (SO_4_^2−^) and nitrogen nitrate (N-NO_3_^−^) are determined by means of a VIS spectrophotometer (DR3900, Hach Lange GmbH, Düsseldorf, Germany) using the LCK353 for SO_4_^2−^ and both the LCK338 and LCK340 cuvette tests for N-NO_3_^−^ (Hach Lange GmbH, Düsseldorf, Germany). N-NO_3_^−^ is determined by the photometric measurement of 4-nitro-2.6-dimethylphenol after reaction with 2.6-dimethylphenol in a solution containing sulfuric and phosphoric acids. SO_4_^2−^ is determined by the photometric measurement of turbidity after reaction with barium chloride. A total of 200 L of agricultural digestate were used as trickling liquid and inoculum for the process. The digestate needed to be diluted with water (volumetric dilution rate 1_digestate_:3.5_water_) in order for the VIS-spectrometric sensor to be able to carry out the measurements. The biogas stream loaded with H_2_S was pumped directly from the gas space of the agricultural digester into the bottom part of the BTF by a membrane pump (N816KT.29DC-B, KNF Neuberger GmbH, Freiburg-Munzingen, Germany). The H_2_S concentration was increased during the last 23 days of operation using H_2_S from a separate gas cylinder connected to the biogas pipes (99.5% H_2_S, Westfalen AG, Münster, Germany). Both gases were mixed in a static mixer prior to entry in the pilot plant. The digestate flow was controlled by a magnetic inductive flow sensor (SM8000, ifm GmbH, Essen, Germany) and was regulated between 85 Lh^−1^ and 250 Lh^−1^. The biogas flow rate was controlled by means of a standard variable area (VA) rotameter (H250/RR/M40/ESK, Krohne Messtechnik GmbH, Duisburg, Germany). The gas stream flowed upwards inside the column, which was filled with the previously mentioned packing material. The gas flow was varied between 0.5 and 0.7 m³h^−1^. Countercurrent to the biogas flow, digestate was pumped into the upper part of the BTF (BN 1-6L, Seepex GmbH, Bottrop, Germany), where it trickled downwards. Both fluids met inside the column, where the absorption and degradation of H_2_S took place. The desulfurized biogas flowed through the upper part of the column back to the agricultural digester, and the digestate into the reactor.

The components in the biogas were measured before and after passing the BTF. CH_4_ and CO_2_ were measured by infrared absorption, and H_2_S was measured electrochemically. Both measurements were carried out in the same gas analyzer in a 12 min cycle (SWG 100, MRU GmbH, Fuchshalde, Germany).

### 2.1. Process Performance Assessment

The efficiency of the process was quantified by the following performance parameters: the removal efficiency (RE), the inlet load (IL), the elimination capacity (EC), the trickling liquid velocity (TLV) and the empty bed residence time (EBRT). These performance parameters are described as follows [13]:

(1) $$RE=\frac{C_{H2S,in}-C_{H2S,out}}{C_{H2S,\;in}}\times 100\;\;\;\;\;\;\;\;\;\;\;\;\;\;\;\;\;\;\;\;\;\;\;\;\;\;\;\;\;\;\;\;\;\;\;\;\;\;[\%]$$

(2) $$IL=C_{H2S,in}\times \frac{{\dot{V}}_{biogas}}{V_{packed\;bed}}\;\;\;\;\;\;\;\;\;\;\;\;\;\;\;\;\;\;\;\;\;\;\;\;\;\;\;\;\;\;\;\;\;\;\;\;\left[\frac{gS-H_{2}S_{}}{m_{}^{3}h}\right]$$

(3) $$EC=\left(C_{in,H2S}-C_{out,\;H2S}\right)\times \frac{{\dot{V}}_{biogas}}{V_{packed\;bed}}\;\;\;\;\;\;\;\;\;\;\left[\frac{gS-H_{2}S_{}}{m_{}^{3}h}\right]$$

(4) $$TLV=\frac{{\dot{V}}_{digestate}}{A_{BTF}}\;\;\;\;\;\;\;\;\;\;\;\;\;\;\;\;\;\;\;\;\;\;\;\;\;\;\;\;\;\;\;\;\;\;\;\;\;\;\;\;\;\;\;\;\;\;\;\;\;\;\;\;\;\;\;\;\;\;\;\left[\frac{m}{h}\right]$$

(5) $$EBRT=\frac{V_{packed\;bed}}{{\dot{V}}_{biogas}}\;\;\;\;\;\;\;\;\;\;\;\;\;\;\;\;\;\;\;\;\;\;\;\;\;\;\;\;\;\;\;\;\;\;\;\;\;\;\;\;\;\;\;\;\;\;\;\;\;\;\;\;\;\left[min\right]$$

where ${\dot{V}}_{biogas}$ represents the gas flow rate (m^3^h^−1^), V_packed bed_ is the fixed packed bed volume (m^3^), C_H2S,in_ and C_H2S,out_ are the H_2_S inlet and outlet concentrations in the biogas (gm^−3^), ${\dot{V}}_{digestate}$ (m³h^−1^) corresponds to the liquid recirculation flow rate and A_BTF_ (m²) is the BTF cross sectional area.

The stoichiometry governing the occurring reactions in the process can be summarized as follows [21]:

(6) $$5H_{2}S+2NO_{3}^{-}\to 5S^{o}+4H_{2}O+2OH^{-}+N_{2}$$

(7) $$5H_{2}S+8NO_{3}^{-}\to 5SO_{4}^{2-}+4N_{2}+4H_{2}O+2H^{+}$$

From these equations, it can be noted that different sulfur and nitrogen molar ratios in the feed result in different product outcomes. This ratio is summarized as the N/S molar ratio and is defined in the following Equation (9). Since the dosage of potassium nitrate (KNO_3_) was carried out discontinuously, the N/S molar ratio in this study refers to the initial value at the moment of dosage.

In order to estimate the nitrate consumption rate, nitrate dosage was carried out manually according to the measured H_2_S value in the biogas. Nitrate concentration was measured from a sample taken from the reactor on a daily basis. The nitrate consumption rate is given by the following Equation (8).

(8) $$\frac{N}{S}ratio=\frac{\left(\left(r_{N-NO_{3}^{-}}\right)*V_{washing\;liquid}\right)/M_{N}}{\left(EC*V_{BTF}\right)/M_{S}}\;\;\;\;\;\;\;\;\;\;\;\;\;\;\;\;\;\;\;\;\;\;\;\;\;\;\;\;\;\;\;\;\;\;\;\;\;\;\;\;\;\;\;\;\;\;\;\;\;\;\left[\frac{mol_{N}}{mol_{S}}\right]$$

(9) $$r_{N-NO_{3}^{-}}=\frac{∆C_{N-NO_{3}^{-}}}{∆t}\;\;\;\;\;\;\;\;\;\;\;\;\;\;\;\;\;\;\;\;\;\;\;\;\;\;\;\;\;\;\;\;\;\;\;\;\;\;\;\;\;\;\;\;\;\;\;\;\;\;\;\;\;\;\;\;\;\;\;\;\;\;\;\;\;\;\;\;\;\;\;\;\;\;\;\;\;\;\;\;\;\;\;\;\;\;\;\;\left[\frac{mg_{N-NO_{3}^{-}}}{\left(L*h\right)}\right]$$

where *M_N_* and *M_S_* correspond to the molar masses of nitrogen and sulfur, respectively. The trickling liquid refers to the digestate used for the operation of the pilot plant, which was recirculated.

### 2.2. Pilot Plant Operation 

The pilot plant was operated for 149 days. During this time, IL and EC, the oxygen concentration in the biogas and the nitrate depletion rate were analyzed, in order to determine their influence on BTF performance. The complete experimental phase includes the start-up time, during which the bacterial cultivation took place. The plant was initially intermittently operated, in order to prevent condensate accumulation in the pipes and damaging of the measurement technology. An overview of all operating conditions throughout the experimental phase are seen in Table 1.

From day 1 to day 36, the pilot plant was operated daily for 9 continuous hours, after which the pilot plant shut down automatically. This was followed by a 14-day period, where the pilot plant was operated daily for 12 continuous hours. For the following 5 days, the plant was operated daily for 16 continuous hours, after which came a period of 24 h operation for 12 days. At the end of this operation period (day 71), the inoculation was considered to have been finalized. From day 72 onwards, the pilot plant was operated continuously. On day 72, condensation containers were installed in the pilot plant and continuous operation was possible.

Before the pilot plant operation began, 1 m³ of digestate was taken from the agricultural biogas plant and was stored for later use. Afterwards, 200 L were diluted with water in a 1:3.5 volumetric proportion and were used in the pilot plant as trickling liquid. From day 1 until day 99, the pilot plant was operated with the mentioned solution. On day 99, a new sample of the stored digestate was taken and diluted in a 1:7.5 volumetric proportion and was used as trickling liquid.

KNO_3_ was added to the stirred tank reactor as an electron acceptor approximately two times per week. The feeding regime can be taken from the following Table 2.

The results presented in this study correspond to daily mean values. During the complete experimental phase, no temperature or pH control was undertaken. The pilot plant was not operated during weekends from day 1 until day 126, after which the plant ran continuously, including weekends, until the end of the experimental phase on day 149. Six different pilot plant operation modes were tested. Each operation mode refers to the number of hours the pilot plant ran before shutting down automatically.

During the first 126 days of operation, no additional H_2_S was added into the biogas stream coming from the agricultural digester. On day 127, the H_2_S concentration in the biogas was artificially increased by dosing pure H_2_S from a gas cylinder.

### 2.3. Microbial Analysis

The microbial community was profiled by 16S rRNA genes analysis. A sample of the packing material was taken on day 106 from the upper half of the BTF and sonicated using an ultrasonic bath (40 KHz, 10 min) to detach the biofilm. The suspended biomass was then centrifuged (10,000× *g*, 10 min) and harvested. The genomic DNA was extracted using DNeasy PowerSoil Pro Kit (Qiagen, Germany) and used as input material at the company STAB VIDA (Caparica, Portugal). The Illumina 16S Metagenomic Sequencing Library preparation protocol with a specific set of primers targeting the V3 - V4 variable regions of the 16S rRNA gene (341F and 785R) was used to perform the library construction. The MiSeq Reagent Kit v3 in the lllumina MiSeq platform using 300 bp paired-end sequencing reads was used to sequence the generated DNA fragments (DNA libraries). The bioinformatics analysis of the generated data was carried out using QIIME2 v2022.2 [22]. The reads were denoised using the DADA2 plugin [23] which trimmed and truncated low-quality regions, dereplicated the reads and filtered chimeras. These reads were subsequently organized in operational taxonomic units (OTUs) and then classified by taxon using a fitted classifier trained using the SILVA (release 138 QIIME) database, with a clustering threshold of 99% similarity. Only OTUs containing at least 10 sequence reads were considered significant. The number of reads in each OTU was represented as relative abundance using the visualization tool Krona [24].

### 2.4. Nutrient Medium and Culture

The digestate and biogas used in this study were the products of the anaerobic digestion of cattle manure, food and fat residues from the food industry. In order to avoid the clogging of the digestate pumps, it was first filtered with a sieve (mesh width = 0.5 mm). No additional bacterial cultures or additional nutrients were added to the medium prior to, during or after the experimental phase. The composition of the undiluted, raw digestate can be taken from Table 3.

## 3. Results and Discussion

### 3.1. Impact of Operating Conditions on RE

**Figure 2:**
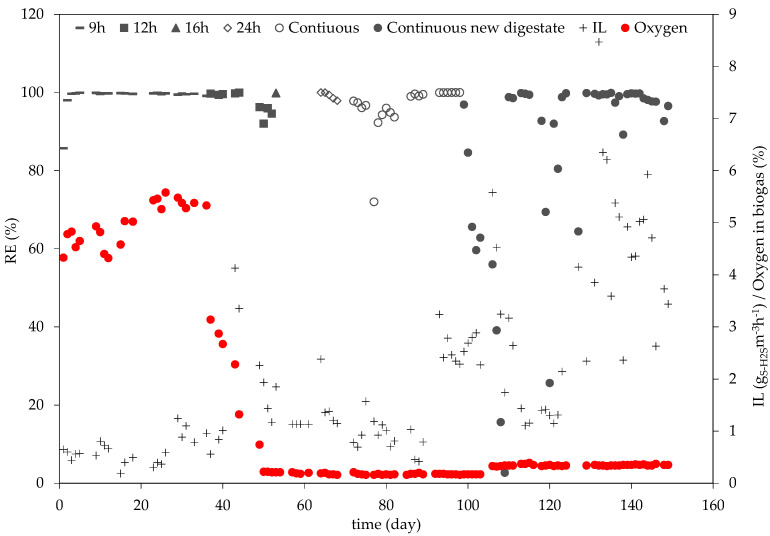
The course of RE and IL throughout the complete experimental phase.

As can be seen in Figure 2, the inlet H_2_S concentrations of the biogas coming from the digester vary strongly over time, which can be identified from the differences in IL. These variations are due to changes in the H_2_S concentration coming from the digester. The agricultural digester uses different substrates to produce biogas, including manure, grass silage and residues from the food industry. The composition of the biogas changes based on the type of substrate being fermented in the digester. The lowest IL was 0.2 gS-H_2_Sm^−3^h^−1^ and was measured on day 15 of operation, and the highest IL (without additional dosage of H_2_S) was measured on day 105 and was 5.6 gS-H_2_Sm^−3^h^−1^. The highest IL throughout the whole study was 8.5 gS-H_2_Sm^−3^h^−1^ and was achieved on day 132. The corresponding RE values to the varying IL are also showcased on Figure 2.

Within the first day of operation, RE went from 85.7% to 98%, and it achieved 99.7% on its third day. The removal efficiency remained above 99% for the entire 9 h operation period. On day 2 and day 16, 17.5 and 50g of potassium nitrate (KNO_3_) were added into the reactor, respectively. The H_2_S concentration in the biogas remained between 19 and 136 ppm_V_, resulting in an initial N/S molar ratio of 30.1 and 48.1 mol mol^−1^. These superstoichiometric conditions were chosen in order to guarantee sufficient nutrient availability in the medium. The mean H_2_S concentration during this phase was 65.2 ± 24.6 ppm_V_. These low H_2_S concentrations could explain the high RE values during the first couple of days of operation. High RE within the first days of the operation of biological desulfurization processes have also been reported in the literature. Sampere et al. [25] operated two suspended biological bioreactors (SBB) for the desulfurization of biogas under anoxic conditions and achieved an RE of 96 and 89% within the first two days of operation, despite having a higher IL than that at the beginning of operation in this work. 

During the 9 h testing period, a mean daily oxygen concentration in the biogas of 4.9 ± 0.43% was measured. Since the pilot plant was operated with biogas coming from a functioning agricultural digester, it is probable that during this time air was temporarily added into the digester as a consequence of maintenance work, leading to an elevated oxygen concentration. On day 11 and day 24, the dissolved oxygen concentration was measured, and it was 2.7 mgL^−1^ and 2.3 mgL^−1^, respectively. These measurements indicate aerobic conditions, also contributing to the high removal efficiencies.

From day 37 to day 52, while the plant was operated for 12 h, the mean H_2_S concentration was 253.3 ± 141.4 ppm_V_. On days 39, 43, 46 and 50, 50 g of KNO_3_ were added. On day 51, 40.1 g KNO_3_ were added to the reactor, whereas on day 52, 26 g of KNO_3_ were added. The initial N/S molar ratio fluctuated between 4.31 and 28.22 mol mol^−1^. Under these conditions, the RE varied between 92% and 100%. The latter RE was measured at a H_2_S concentration of 450 ppm_V_ on day 44. At this time, the percentage of oxygen in the biogas had decreased to 1.32%. Although this concentration had significantly decreased compared to the 9h operation period, these conditions could also have favored aerobic bacteria, contributing to the high H_2_S RE. Due to a gas leakage in the pilot plant, from day 45 until day 48, the plant had no biogas flow. The BTF was fed with trickling liquid so as to sustain the microbial community until the leakage was repaired. On day 49, operations resumed. The oxygen percentage in the biogas had by then decreased to 0.35%. From day 49, the RE also decreased to 96.2%, even reaching 92% on day 50, and then slowly increased again, reaching 94.6% on day 52. Day 50 marks the day on which the oxygen concentration in the biogas stabilized for the rest of the experimental phase. On day 51, the oxygen concentration in the biogas had decreased to 0.22%, and, by this point, the dissolved oxygen concentration in the trickling liquid was 0.63 mgL^−1^. The decrease in oxygen concentration in both the biogas and the trickling liquid indicates a slow transition into anoxic conditions, which could explain the fluctuation in RE during the last days of the 12h operation period.

From day 53 until day 58, the plant was operated for 16 h. On day 54, the H_2_S sensor in the gas analyzer was damaged, and no H_2_S measurements were possible until day 64. Only on day 53 was it possible to carry out a H_2_S measurement. The mean RE value for day 53 was 99.89%, with a mean H_2_S concentration in the biogas of 214.12 ± 35.2 ppm_V_. During this operation period, the O_2_ concentration in the biogas decreased to 0.21%. On days 57 and 58, 100.3 and 71.8g of KNO_3_ were added to the reactor.

For the following 13 days (59 – 71), while the plant was operated for 24 h, the mean H_2_S concentration was 158 ± 43.5 ppm_V_. The mean RE was 99.1%, reaching a maximum of 100% on days 64 and 65, with H_2_S concentrations of 242.1 and 146 ppm_V_, respectively. The minimum RE determined during this period was 97.7% on day 68. On days 64, 65, 66 and day 67, 30 g, 50 g, 10 g and 110 g of KNO_3_ were added to the reactor, keeping the initial N/S molar ratio between 9.8 and 19.7 mol mol^−1^. The mean oxygen percentage in the biogas was 0.19%, which favors anoxic conditions.

From day 72 until day 98, the plant was operated continuously. The mean RE value during this period was 96.4 ± 6.08%, with a mean H_2_S concentration of 154.7 ± 87.0 ppm_V_. The mean oxygen percentage in the biogas was 0.17 ± 0.012%. On day 92, the dissolved oxygen concentration in the trickling liquid was 0.15mgL^−1^, indicating anoxic conditions. These RE results are consistent with the work of Zeng et al. [17], who operated an anoxic BTF using digestion slurry and biogas from rural household biogas digesters. In the aforementioned study, an average RE of 94.4 ± 4.37% was achieved in a BTF packed with polyurethane foam. Although their IL showed smaller variations, the RE fluctuated in a similar manner to the one seen in this work. 

Due to an increase in the turbidity of the trickling liquid and difficulties in measuring the relevant parameters, on day 99, the digestate in the reactor was fully replaced. The raw digestate was diluted with water in a higher dissolution proportion (1:7.5) than at the beginning of the experimental phase. The packing material in the BTF was not replaced, thereby maintaining the formerly cultivated biofilm. From day 99 until day 126, the mean H_2_S percentage in the biogas was 240 ± 124.9 ppm_V_. The RE ranged from 99.99% to 2.7%. The lowest RE was measured on day 109, 8 days after the exchange of the digestate. From day 127 until the last day of operation (day 149), the H_2_S percentage in the biogas was increased, with a mean H_2_S percentage of 491.9 ± 146.3 ppm_V_. The RE ranged from 99.9% to 92.7%, with a mean value of 96.4%. The initial N/S molar ratio was kept between 0.28 and 8.24 mol mol^−1^. On day 118, the RE dropped to 92.73%, and it continued to drop the next day to 69.41%. On day 120, the RE dropped further to 25.63%, even though the IL did not increase. Since no further explanation for the RE decrease could be found, it was assumed that the intermixing between the gas and trickling liquid had become insufficient. Therefore, on day 121, the TLV was increased from 1.5 mh^−1^ on average to a mean value of 4.5 ± 0.21mh^−1^. After the TLV was changed, an increase in RE was observed. The TLV remained at 4.5 mh^−1^ until the end of the experimental phase.

The mean oxygen concentration in the biogas from day 99 to day 149 was 0.26 ± 0.07%. The dissolved oxygen concentration in the trickling liquid varied between 0.05 mgL^−1^ and 0.38 mgL^−1^. The latter value was measured on day 113. The most frequent dissolved oxygen value was 0.13 mgL^−1^.

### 3.2. Impact of IL and pH on EC, RE and SO_4_^2−^

**Figure 3:**
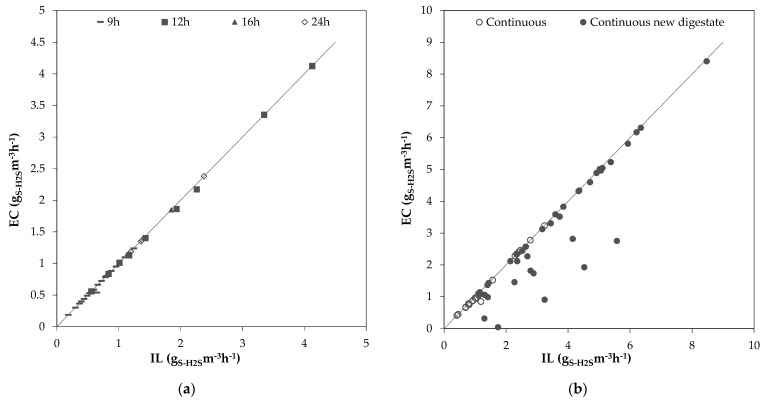
Dependency of EC on IL for (**a**) the 9 h, 12 h, 16 h and 24 h operation periods; (**b**) the first continuous operation period and the continuous operation period with new digestate.

Figure 3 showcases the relationship between IL and EC. The diagonal in the diagram represents a removal efficiency of 100%. All daily mean values of EC vs IL, which were determined during the 9 h, 12 h, 16 h and 24 h operation periods (Figure 3a) and the two continuous periods (Figure 3b), can be seen. The highest IL tested in this study was 8.47 gS-H_2_Sm^−3^h^−1^ with an EC of 8.4 gS-H_2_Sm^−3^h^−1^ (RE 99.1%), achieved on day 132. Sempere et al. [25] achieved an EC of 10.63 gS-H_2_Sm^−3^h^−1^ at an IL of 13.37 gS-H_2_Sm^−3^h^−1^ (RE 90.7%) in an SBB, and Khanongnuch et al. [18] achieved an EC of 19.2 gS-H_2_Sm^−3^h^−1^ with a RE of 98.2% in a 2.11 L BTF filled with polyurethane foam cubes. While the IL in this study was lower than the IL of the aforementioned authors, the H_2_S concentrations of all three studies were similar (ca. 500 ppm_V_), making them comparable. The higher RE achieved in this study can be attributed to a longer EBRT of 8.8 min compared to 5.5 min [25] and 3.5 min, possibly allowing a better mixing with the washing liquid.

The pH value was monitored during all operation periods. No adjustments of the pH value were carried out, except on day 142, where the automatic regulation of pH was tested but not further implemented. On day 1 of the operation, the pH was 7.2, and it decreased to 6.6 on day 2. It remained within the range of 6.9 to 6.5 until day 87, when the pH decreased to 4.6. On the following day (day 88), the value had increased to 5.8, and, on day 89, it was 6.6. From day 89 until day 99, the pH value remained at 6.4 ± 0.09. On day 99, the trickling liquid was fully replaced and the pH value dropped to 5.9, where it remained until day 113. After day 113, the pH slowly and steadily increased until it reached a value of 6.8 on the last day of operation. The following Figure 3 shows the course of the pH value throughout the complete experimental phase, as well as the corresponding IL.

The dependency of H_2_S absorption on pH has been extensively discussed in the literature [26,27]. The absorption process benefits from high pH values, as they contribute to the dissociation of H_2_S to HS^−^ and H^+^ in the liquid phase, allowing higher concentrations of H_2_S to be absorbed [28]. Higher pH levels, close to 8, also benefit microbial activity and growth. However, some authors recommend a pH of 6.8 during the bacterial culture phase, in order to minimize sulfide accumulation [29].

**Figure 4:**
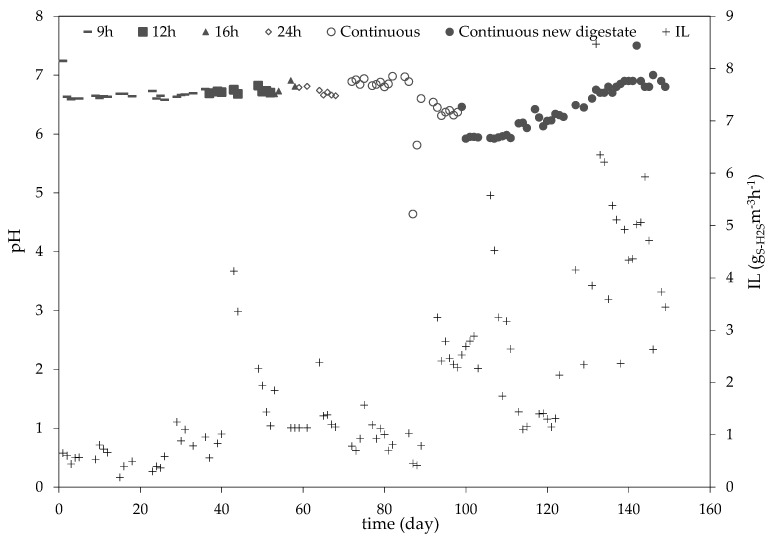
pH and IL progress throughout the experimental phase.

In this study, the pH was not regulated, only monitored, in order to test the robustness of the removal efficiency with respect to abrupt naturally occurring changes in pH. As can be seen in Figure 4, the pH remained mostly constant between days 2 and 86, ranging from 6.5 to 6.9.

On day 43 and 44, IL went up by 3.1 gS-H_2_Sm^−3^h^−1^ on average compared to the previous operation days. The pH remained unchanged at a mean value of 6.7 during the two days of higher IL, as well as later. The RE also remained above the mean values of the previous days, above 99%.

The sudden drop in pH on day 87 was caused by a spontaneous spike in H_2_S that lasted for one hour. The H_2_S concentration in the biogas reached 950 ppm_V_. Up to day 86, the mean H_2_S concentration in the biogas was 121.7 ± 90.7 ppm_V_. Even though the pH value fell significantly during the two days, the RE was not affected, staying above 99%.

The fact that pH remained low during the following days after the spike is probably due to the accumulation of HS^−^ and H^+^ ions from the dissociation of H_2_S and not from the conversion of H_2_S to (SO_4_)^2−^ and H^+^. pH stability in anoxic desulfurization processes without any regulation has been reported in literature. Sempere et al. [25] and Zeng et al. [17] also observed pH values between 5.5 and 6.8 and 6 and 8, respectively, without the use of automatic regulation. Both of these authors operated their plants (SBB and BTF respectively) with real municipal biogas digestate.

**Figure 5:**
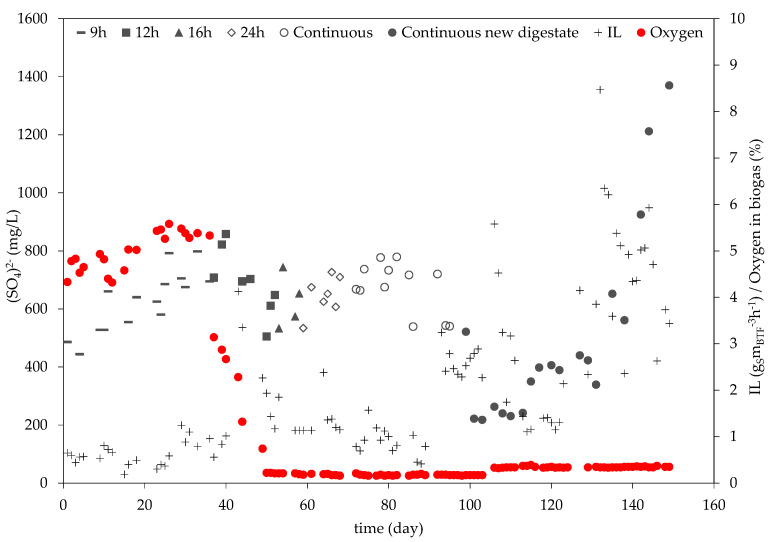
(SO_4_)^2−^ and IL progress throughout the experimental phase.

In Figure 5 below, it can be seen that there was no equivalent abrupt increase in (SO_4_)^2−^ on day 87 or in the following days. The week prior to the H_2_S spike in the biogas, the (SO_4_)^2−^ concentration had a mean value of 703 mgL^−1^, whereas, the week afterwards, the (SO_4_)^2−^ even decreased to 585.5mgL^−1^.

Although the increase in the H_2_S concentration on days 43 and 44 lasted longer than the spike in H_2_S on day 87, the change in pH was much smaller. This may be due, on the one hand, to the fact that the digestate on days 43 and 44 was fresher, possibly presenting a higher buffer capacity. On the other hand, during days 43 and 44, the plant was being operated under aerobic conditions, allowing for a microbial culture to adapt and be able to degrade H_2_S, preventing HS^−^ ion accumulation. 

As can be seen in Figure 5, (SO_4_)^2−^ formation fluctuated throughout the experimental phase. During the first 40 days of operation, a constant increase in the (SO_4_)^2−^ concentration can be observed. This behavior is expected, as H_2_S is gradually degraded by the bacterial consortium and accumulates in the trickling liquid. On day 40, a maximum (SO_4_)^2−^ concentration of 858 mgL^−1^ was measured, after which it started to decrease. The decrease pattern coincides with the steady decrease of the oxygen concentration of the biogas, pointing to an adjustment of the bacteria to the new oxygen-limited conditions. From day 1 until day 36, the oxygen percentage in the biogas was on average 4.5%. On day 37, the oxygen percentage decreased to 3.14% and continued to decrease until day 50, when it stabilized at 0.21%, where it remained until the end of the experimental phase. These fluctuations of electron acceptor availability can cause a shift in the microbial community, possibly contributing to the cultivation of (SO_4_)^2−^ reducing bacteria. This would explain why, from day 40 until day 99, the (SO_4_)^2−^ concentration fluctuated between 858 mgL^−1^ and 505 mgL^−1^, without showing an increasing tendency. From day 99 until the end of the experimental phase, the (SO_4_)^2−^ concentration increased as the IL increased. This can be interpreted to mean that the microbial community adapted to the anoxic conditions and the expected (SO_4_)^2−^ accumulation took place. The highest (SO_4_)^2−^ concentration was reached on day 149 and was 1370 mgL^−1^.

### 3.3. Impact of Operating Conditions on Nitrate Depletion Rate

**Figure 6:**
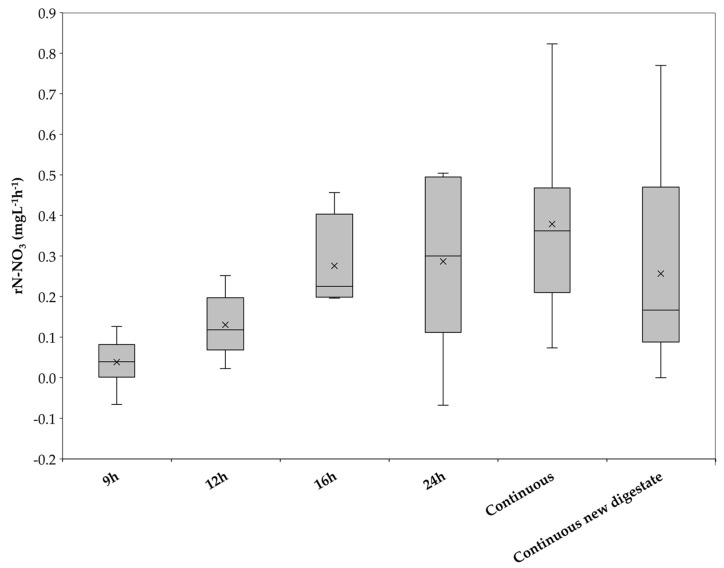
Box plot of N-NO_3_ depletion rates for all operation periods during the experimental phase.

Nitrate depletion can be understood as a measure of process efficiency. The faster nitrate is depleted, the higher the microbial activity and removal efficiency. During the six periods of plant operation, different nitrate depletion rates were established, as can be seen in Figure 6. In this figure, the nitrate depletion rates were represented in a box plot. The horizontal line inside each box corresponds to the median value of each operation period, and the cross to the average value [30].

Figure 6 shows that, during the 9 h period, the mean nitrate depletion rate was the lowest of the whole experimental phase, averaging 0.04 mgN-NO_3_^−^ L^−1^h^−1^. This result is expected since, during the first days of operation, the bacterial community had not yet adapted, and also due to the presence of oxygen in the system. The lowest result even has a negative value of −0.07 mgN-NO_3_^−^L^−1^h^−1^, indicating that there was no consumption, but rather a production of nitrate. This is consistent with the high oxygen concentration in the biogas, leading to the aerobic conditions in which the plant was operated during the 9 h operating period. It is possible that ammonia was converted into nitrate. The following 12 h operating period showed higher nitrate depletion rates than the previous period. Although aerobic conditions still governed the plant at the beginning of the 12 h period, over the course of the 16 days of operation, the oxygen in the biogas decreased, until it stabilized at 0.21% on the last three days. These three days also saw an increase in the nitrate depletion rate from an average of 0.09 mgN-NO_3_^−^L^−1^h^−1^ in the previous days to that of 0.21 mgN-NO_3_^−^L^−1^h^−1^ during the last three days. 

The depletion rate continued to increase during the following operating periods, reaching a maximum of 0.82 mgN-NO_3_^−^L^−1^h^−1^ on day 94 during the continuous operation of the pilot plant. As previously mentioned, on day 99, the trickling liquid was replaced. This led to a significant decrease in the nitrate depletion rate, which went from 0.44 ± 0.2 mgN-NO_3_^−^L^−1^h^−1^ the week prior to a mean value of 0.1 ± 0.08 mgN-NO_3_^−^L^−1^h^−1^ over the following 11 days. This shows an inhibition of the microbial activity, probably due to the new conditions caused by the exchange of trickling liquid. Another possible explanation for the decrease in nitrate depletion rate is that the adapted biomass dispersed in the trickling liquid was lost after it was replaced. The mean nitrate depletion rate from day 115 until the end of the experimental phase (day 149) was 0.35 ± 0.21 mgN-NO_3_^−^L^−1^h^−1^, indicating an adaptation or growth of the microbiology to the fresh trickling liquid.

Poser et al. [31] operated a two-stage desulfurization plant using cellular concrete filtration followed by a BTF with a limited oxygen concentration. The used biogas mimic contained an oxygen concentration between 0.5 and 0.8%, which is higher than but comparable to the concentration in this study. Although the authors did not operate the BTF with other electron acceptors besides the oxygen present in the biogas, the reported RE were higher than 95% (with an IL of 5 gm^−^³h^−1^). This may indicate that the aerobic desulfurization of biogas can occur even under oxygen-limited conditions. However, based on the measured nitrate degradation throughout the experimental phase of this work, it could be postulated that H_2_S removal took place under both anoxic and aerobic conditions.

The following Table 4 shows a comparison of different nitrate degradation rates under similar experimental conditions. It can be seen that the highest nitrate degradation rate of this work is similar to the degradation rates found in [18,32]. The experimental conditions of [21] show that the influence of the initial concentration of nitrate in the trickling liquid has a significant influence on the nitrate degradation rate.

This observation becomes especially apparent when compared with the degradation rates of [29,33]. Both are considerably higher than those of this work, possibly resulting from initial nitrate concentrations being between 23.2 and 124.3 times higher. A much higher IL than that of this work can also be noted, further contributing to the higher nitrate depletion rates.

The influence of nitrate concentration could also have an impact on RE. Zeng et al. [17] reported a decrease in RE as the nitrate concentration dropped below 45 mgL^−1^. Soreanu et al. [34] also states that maintaining a nitrate concentration of 200 mgN-NO_3_^−^L^−1^ is ideal to guarantee a high RE in a BTF being operating with an IL of 4 gS-H_2_Sm^−3^h^−1^. 

### 3.4. Impact of the Microbial Community on the Overall BTF Performance 

The microbial community present on the BTF on day 107 was analyzed by 16S rDNA amplification. The sample generated 211,002 raw sequencing reads, which is in accordance with the expected output (≈ 100,000 sequence reads). The mean read length was 300 bp. After denoising, 414 unique OTUs were identified. Diversity indexes (Simpson, Shannon and Chao1) were calculated to evaluate the bacterial species’ richness and evenness. The values of the Simpson (0.969), Shannon (4.397) and Chao1 (305) indexes indicated the lack of dominant microorganisms and the high diversity of the OTUs present in the sample. This wide variety of OTUs can be explained by the complex composition of the trickling liquid composed of diluted digestate (Table 2).

**Figure 7:**
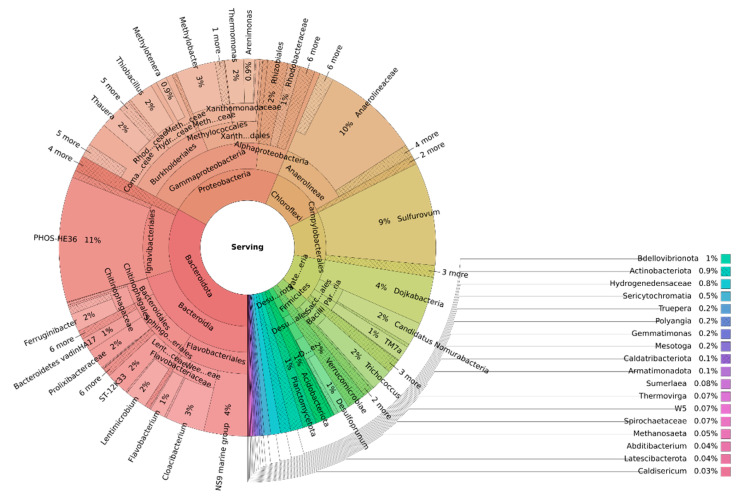
Sunburst chart analysis showing the family-level relative abundance in the BTF on day 106.

A sunburst chart analysis was used to depict the family-level relative abundance of the microbial sample characterized (Figure 7). 

The rings represent different levels in the phylogenetic tree. The most abundant phylum was *Bacteroidota* (33%), which was mainly represented by the orders *Ignavibacteriales* (11%), *Flavobacteriales* (9%), *Sphingobacteriales* (5%), *Bacteroidales* (4%) and *Chitinophagales* (3%). Bacteria included in this phylum are mostly anaerobic, although some are facultatively anaerobic and saccharolytic, although proteins and other substrates may be utilized [35]. The dominance of this phylum may be attributed to the organic matter content of the digestate used, which may lead to the growth of these bacteria. The most representative bacteria of this phylum were the PHOS – HE36 (11%) and NS9 marine (4%) groups, followed by the genera *Cloacibacterium* (3%) and *Lentimicrobium* (2%). The dominance of the phylum *Bacteroidota* and the presence of the PHOS – HE36 group has been recently reported in a sequencing batch reactor treating a digestate of food waste rich in ammonium and salts [36]. The *Lentimicrobium* genus was also reported in an anoxic bioreactor desulfurizing biogas using nitrified landfill leachate as electron acceptor source, indicating the tolerance of this genus to significantly dissolved sulfide concentrations [16]. 

The phylum Proteobacteria (23%) was the second most abundant phylum in the biofilm. It was mainly represented by the *Gammaproteobacteria* class and included heterotrophic denitrifiers such as *Comamonadaceae* (3%) and *Thauera* (2%) [37]. *Thiobacillus* was the most abundant genus within this phylum capable of oxidizing reduced sulfur compounds. This genus has been widely reported in other bioreactors to perform the anoxic desulfurization of biogas using either synthetic media [38] or biogas slurry [39] as a trickling solution. It is remarkable that the presence of bacteria widely reported to be related to the anaerobic oxidation of methane, such as the genera *Methylobacter* (3%) and *Methylomonas* (1%), was promoted by the feeding of real biogas [40]. 

The phylum *Chloroflexi* (11%) was mainly represented by the family *Anaerolineaceae* (10%), widely known to be chemoheterotrophs [41].

**Figure 8:**
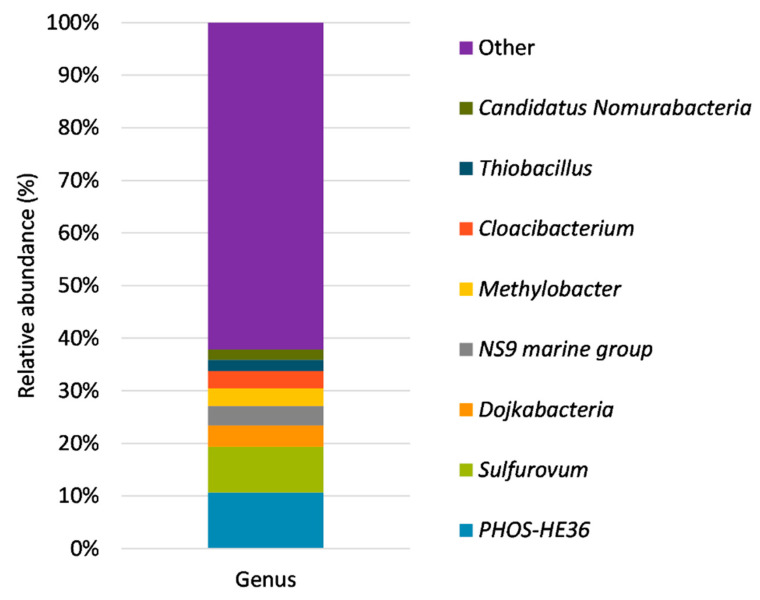
Relative abundance at genus level of the microbial consortium present in the BTF on day 106.

Most bacteria capable of oxidizing reduced sulfur compounds belong to the *Campilobacterota* (formerly known as ε-proteobacteria) (10%) phylum. This phylum was mainly represented by the genus *Sulfurovum* (9%), the second most abundant genus present in the sample (Figure 8).

This genus was followed by the genera *Sulfuricurvum* (0.5%), *Sulfurimonas* (0.2%) and *Arcobacter* (0.2%). These genera are commonly found in bioreactors performing the anoxic desulfurization of biogas. Xu et al. [42] considered *Sulfurovum* to be the main genus involved in the autotrophic removal of sulfide in a suspended growth bioreactor fed with synthetic wastewater rich in nitrate. Other genera like *Sulfurimonas* and *Arcobacter* were found to be dominant in an anoxic gas-lift bioreactor desulfurizing biogas using nitrified landfill leachate as an electron acceptor [16]. The genus *Sulfuricurvum* has been considered an aerobic sulfide oxidizer by Das et al. [2]. These authors found this genus simultaneously removing H_2_S and NH_3_ from raw biogas in a hollow fiber membrane bioreactor. The presence of this genus can be explained by the oxygen content of the biogas, which ranged in value up to 5.6% throughout the operation. 

Finally, it is important to stress the presence of the phylum *Desulfobacterota* (2%), whose main representatives belonged to the genera *Desulfoprunum* (0.8%) and *Desulfobacterium* (0.4%). Both genera have been reported to be anaerobic sulfate-reducing bacterium capable of proliferating in diverse liquid effluents such as wastewater or landfill leachates [43]. The presence of this type of bacteria explains the fluctuation of the concentration of (SO_4_)^2−^ mentioned in the previous sections. Since the pilot plant was mostly operated using biogas under anaerobic conditions, these bacteria were able to establish and metabolize (SO_4_)^2−^, hindering the increase of (SO_4_)^2−^ concentration in the trickling liquid.

## 4. Conclusions

The performance of a pilot-scale BTF for biogas desulfurization under anoxic conditions using real biogas and employing diluted agricultural digestate as trickling liquid was tested in this study. The results showed that diluted agricultural digestate is suitable as trickling solution for the anoxic desulfurization of the biogas. The operating conditions used in this study, in which no pH or temperature regulation was used, reveal that the usage of a BTF is a valid option for biogas desulfurization. Specifically, the usage of agricultural digestate as trickling liquid proved that it harbors relevant bacteria strands that can be cultivated and are able to oxidize H_2_S from the biogas. The highest removal efficiency achieved under anoxic conditions was 99.8% with an IL of 5.02 gS-H_2_Sm^−3^h^−1^ (541.7 ppm_V_), whereas the highest EC was 8.4 gS-H_2_Sm^−3^h^−1^ (RE 99.4%). Nitrate degradation was also possible while desulfurization was taking place, with 0.81 mg N-NO_3_^−^L^−1^h^−1^ being the highest value. These results have a significant impact on how biogas desulfurization can be conducted in the agricultural industry. The fact that diluted digestate can be used in an innovative way for the desulfurization of biogas not only contributes to a circular economy in the agricultural sector by reintroducing sulfur and other sulfur compounds into the crops cycle, but also by possibly lowering operational costs, since the use of additional expensive chemicals for biogas desulfurization (such as amines or solutions for pH regulation) would no longer be necessary.

## Figures and Tables

**Table 1:** Operating conditions during experimental phase.

Time [d]	Experiment Duration [hours]	KNO_3_ Dosage [g]	EBRT [min]	TLV [m/h]	H_2_S_inlet_ [ppm_V_]	pH
1–36	9	14–50	9.2–18.1	1.3–1.6	19–136	6.5–7.2
37–52	12	50	8.2–13.4	1.4	51–444	6.7–6.8
53–58	16	50	8.91–9.2	1.3–1.4	171–160	6.7–6.9
59–71	24	50	8.8–11.13	1.2–1.3	124–300	6.7–6.8
72–98	continuous	50–150	8.8	0.7–3.4	63–600	4.6–7
99–149	continuous *	13–500	8.8–11.4	1.4–5.8	250–500	5.9–7.5

* Trickling liquid fully replaced. The same digestate as in the first 22 days was used. Dilution ratio 1:7. Packing material in BTF was not replaced.

**Table 2:** Potassium nitrate dosage regime.

Dosing Days [d]	Experiment Duration [hours]	KNO_3_ Amount [g]
2	9	17.5
16, 23, 29, 36	9	50
39, 43, 46, 50	12	50
51, 52	12	40, 26
53, 57, 58	16	22, 100, 72
59, 64, 65, 66, 67	24	30, 30, 50, 10, 110
72, 80, 82, 85, 88, 89, 92, 95, 96	Continuously	150, 100, 30, 123, 100, 140, 380, 50, 50
99, 110, 117, 120, 122, 124, 127, 131, 135, 138, 142, 144, 149	Continuously *	50, 500, 50, 50, 200, 50, 700, 500, 500, 500, 500, 250, 700

* new digestate.

**Table 3:** Composition of the undiluted digestate.

Parameter	Concentration (mgL^−1^)
pH	7.95
Dry mass (%)	0.54
Organic matter/ residue on ignition	2300
N-NH_4_^+^	410
NO_3_^−^	18.1
N total	490
P_2_O_5_	110
K_2_O	800
MgO	50
CaO	230
Na_2_O	470
S	30
Zn	0.8
B	0.15
Fe	12.5
Cu	0.12
Mn	1.4
Mo	0.07
Co	0.06

**Table 4:** Comparison of different N-NO_3_ depletion rates.

	r_N-NO3-_ [mg*L^−1^*h^−1^]	IL [gS-H_2_Sm^−3^h^−1^]	pH	Initial N-NO_3_^−^ Concentration [mgL^−1^]	Substrate Dosage
[18]	2.26 *	3.5–5.6	7	94.5	Continuous
[32] **	0.52	-	-	50	Continuous
[21]	6.41	202.5*	6.8–7	430	Continuous
[29] *	39.1	117.7	6.2–7.8	2.300	Continuous
[33] ***	46.5 *	7.9	7	48	Continuous
This work’s max. value	0.82	2.41	6.3	18.5	Manual

* mean value; ** no BTF operation. Nitrate depletion rate estimated in double-jacket glass tempered cells with dosage of sodium sulfide. *** continuous dosing of nitrate with varying liquid dilution rates.

## Data Availability

Not applicable.
